# Joint optimization of high-speed train timetables, speed levels and stop plans for increasing capacity based on a compressed multilayer space-time network

**DOI:** 10.1371/journal.pone.0264835

**Published:** 2022-03-03

**Authors:** Angyang Chen, Xingchen Zhang, Junhua Chen, Zhimei Wang

**Affiliations:** School of Traffic and Transportation, Beijing Jiaotong University, Beijing, China; National Taiwan University of Science and Technology, TAIWAN

## Abstract

With the steady increase in passenger volume of high-speed railways in China, some high-speed railway sections have faced a difficult situation. To provide more transport services, it is necessary to add as many trains as possible in a section to increase capacity. To solve this problem, a compressed multilayer space-time network model is constructed with the maximum number of trains that can be scheduled in the train timetable as the objective. The combination of the train stop plan and speed level is represented by the layer of network where the train is located, and constraints such as train selection, train safety, train overtake and cross-line trains are considered. A method based on timing-cycle iterative optimization is designed to decompose the original problem into multiple subproblems, and the solving order of the subproblems is determined by a heuristic greedy rule. Taking the Beijing-Jinan section of the Beijing-Shanghai high-speed railway as an example, the maximum number of trains was increased by 12.5% compared with the timetable before optimization. The saturated timetables provide detailed schedules, which helps decision-makers better adjust the timetable to run more trains.

## Introduction

With the rapid development of high-speed railways worldwide, this good travel experience has attracted an increasing passenger flow, causing the capacity of some busy high-speed railways to approach saturation. To provide more services, operation strategy improvements with existing resources (e.g., modifying timetables) can be applied to increase the capacity (the maximum number of trains that can be scheduled) of the railway system in order to satisfy the higher transport demand.

A typical passenger train scheduling process consists of the following sequential steps: passenger demand surveying, train service plan development, and train timetabling. Under normal circumstances, these three steps are carried out sequentially. However, it should be noted that the speed level and the stop plan of trains are two factors that affect capacity that are determined in the second step (train service plan development). This will result in an inability to adjust the train’s speed level and stop plan in the timetabling process, which finally determines the number of trains. Optimizing each stage of transportation planning individually and in order may be able to obtain the optimal solution of this stage, but for the whole system, it can only achieve a suboptimal solution. Therefore, the purpose of this paper is to find a way to optimize the train timetable, speed level and stop plan at the same time.

A space-time network is a kind of modeling method that is often used to solve railway timetabling problems. First, an abstract train operation network is constructed according to the physical railway network. Then, according to the optimization objectives and constraints, the train selects the arcs in the space-time network to determine the process of train operation in time and space, that is, the train timetable. However, similar to the literal meaning of a space-time network, the typical space-time network contains only two kinds of information: time and space. To achieve the purpose of the joint optimization of the timetable, speed level and stop plan, one important challenge is to express speed and stop information in the network.

To solve this problem, the multilayer space-time (MLST) network is proposed in this paper. For each combination of stop plans and speed levels, a space-time network will be constructed. Therefore, the MLST network is composed of multiple space-time networks. For each train, there will be multiple alternative space-time networks. The train’s choice of a space-time network entails the choice of a speed level and stop plan. Just as a MLST network is composed of multiple space-time networks, the number of arcs in MLST network is several times of that in a space-time network. To reduce the number of arcs in the MLST network to improve the computational efficiency, a network compression technology is proposed according to the characteristics of the timetabling problem to generate the compressed multilayer space-time (CMLST) network. For large-scale real-world problems, the timing-cycle iterative method is used to decompose a large-scale problem into multiple subproblems that can be solved in a short time. Finally, the proposed model and method are verified through the example of the Beijing-Shanghai high-speed railway.

The remainder of this paper is organized as follows: A literature review is presented in Section 2. In Section 3, we first describe the passenger flow section and four aspects that need to be satisfied in the timetabling process. Then, the construction process of the MLST network and the CMLST network is described in detail. Section 4 develops a linear integer programming model with six kinds of constraints. Section 5 describes the proposed timing-cycle iterative method with a greedy rule that is integrated with the Gurobi solver. Section 6 shows the results of a real-world case to verify the validity of the proposed model and method. Finally, conclusions are presented in Section 7.

## Literature review

Optimizing the train timetables is an important transportation organization strategy to increase the capacity. In recent years, many remarkable studies have been devoted to train timetabling problems. Goverde [1] presented a model and an algorithm to compute the propagation of initial delays over a periodic railway timetable. The model proposed by Meng L and Zhou X [2] periodically optimizes schedules for a relatively long rolling horizon while selecting and disseminating a robust meet-pass plan for every roll period. Four fundamental timetable formulations suitable for optimization were defined by Harrod [3]. Corman et al. [4] considered the biobjective problem of minimizing train delays and missed connections to provide a set of feasible nondominated schedules to support this decisional process. Cacchiani and Toth [5] surveyed the main studies dealing with the train timetabling problem in its nominal and robust versions. Niu and Zhou [6] focused on optimizing a passenger train timetable in a heavily congested urban rail corridor. Sun et al. [7] proposed a multiobjective optimization model for train routing on a high-speed railway network, which can provide an important reference for train plans in providing better service. Arenas et al. [8] proposed an alternative mathematical model to address the train timetabling problem (TTP) and presented a genetic algorithm implementing the model to rapidly obtain near-optimal train timetables. Wang et al. [9] proposed an event-driven model that involves three types of events, i.e., departure events, arrival events, and passenger arrival rate change events. Samà et al. [10] and Fu and Dessouky [11] examined various important topics in train scheduling, including timetabling under complex network or N-track conditions and real-time scheduling to mitigate delay propagation under various degrees of disturbances. Hu X et al. [12] found that the high circuity of train paths can be decreased with the construction of a high-speed railway line, which indicates that the structure of the high-speed rail (HSR) network in China should become more complete and that the HSR network can make the Chinese railway network more efficient. Liu S et al. [13] studied the performance and mutual influence of a syncretic railway network (SRN) that comprises high-speed railways, regional railways, and urban rail transit under the condition of traffic overload during peak hours.

As mentioned above, there have been many studies and results on the train timetabling problem. However, there are many stages of train transport planning, such as line planning, timetabling and vehicle scheduling, which depend greatly on one another. One of the weaknesses of the timetabling planning solutions is that it does not optimize the plans of each stage comprehensively, so it can only reach the local optimization. It is therefore beneficial to solve them in an integrated way rather than sequentially. The trend of follow-up research has been to combine the train timetabling problem with other problems. In recent years, many integrated approaches for railway operation optimization have been proposed.

Lusby et al. [14] analyzed the structure of the resulting integer program with respect to decomposition approaches and showed that decompositions that are superior to the canonical decomposition exist in planning stages, line planning, timetabling, and vehicle scheduling. Veelenturf et al. [15] modeled and solved the crew rescheduling problem with retiming. Kaspi M and T Raviv [16] formulated an integrated line planning and timetabling model with the objective of minimizing both user inconvenience and operational costs. Corman and Meng [17] offered a systematic review of real-time train dispatching, rescheduling, and disposition under stochastic and dynamic conditions. Schmidt and Schöbel [18] studied the integrated problem of determining a timetable and passengers’ routes simultaneously. Zhang and Nie [19] proposed a minimum cycle time calculation (MCTC) model based on the periodic event scheduling problem (PESP) for a given train line plan for integrating capacity analysis with timetabling. Burggraeve S [20] proposed a heuristic algorithm to build a railway line plan from scratch that minimizes passenger travel time and operator cost and for which a feasible and robust timetable exists. Schöbel [21] developed and discussed a generic biobjective model for integrating line planning, timetabling, and vehicle scheduling. Shi J et al. [22] proposed an effective method for collaboratively optimizing the train timetable and accurate passenger flow control strategies on an oversaturated metro line.

These studies provide strong support for the joint optimization of train timetables and other issues. The recent publications associated with the integrated optimization of timetabling are listed and compared in Table 1.

**Table 1:** Recent studies on joint train timetabling optimization.

Major decision variables	Model	Algorithm	Publication
Train routing and ordering	Time–space network	Lagrangian relaxation solution framework	Meng and Zhou [23]
Time at which the event takes place; train is canceled or not	Integer programming model	Commercial optimization software CPLEX	Veelenturf et al. [24]
Arrival/departure time; average segment speed	Discretized space-time network to represent variable speed	Commercial optimization software GAMS	Yang et al. [25]
multicommodity variables representing both timetable and speed profile characteristics	Discretized space-time-speed grid network	Dynamic programming	Zhou L et al. [26]

Among the research performed on train timetabling, models based on a space-time network have demonstrated great power. The space-time network representation of the timetabling problem is well-developed since Caprara et al. [27] propose a integer programming based on a directed graph for solving the classic train timetabling problem. Harrod [28] proposed a directed hypergraph formula based on time discrete spatiotemporal network to deal with the conflict between train paths in space-time network, and applied the model to the train timetabling problem at the micro level of busy railways in America.

When the train diagram problem is combined with other problems, the space-time network needs to be extended to consider more variables. Some research have added the "state" dimension to form the space-time-state network modeling method. Zhou et al. [29] proposed a space-time speed network modeling method to achieve the collaborative optimization of train diagram problem and train speed with the goal of minimizing the total travel cost. Xu et al. [30] considered the "state" dimension of space-time state network as the state of locomotive traction train, and built a model to realize the collaborative optimization of train timetable and locomotive turnover. Meng et al. [31] optimized passenger service and train timetable by comprehensively considering passenger demand, infrastructure and vehicle capacity.

Space-time network is also used in the research of public transport and rail transit [32–34]. However, the goal of schedule optimization of public transport and rail transit is to optimize the departure interval at initial station in different periods according to the passenger flow demand. The similarity between these studies and high-speed railway is that they all need to build space-time network. These problems are essentially a network flow problem, and the solution process is to select the arcs in the network. In the application of MLST, the most important difference of high-speed railway vs other transportation is that the relevant constraints of time headway are required to avoid the conflict of trains in time and space. These constraints are the most difficult constraints in railway optimization problems, which will greatly improve the difficulty of finding a feasible solution. Therefore, for the improvement of railway capacity, there is little reference experience in public transport and rail transit.

According to the analysis of literatures, the method based on space-time network shows strong adaptability in railway transportation organization optimization. However, for the increasing of railway capacity, it is necessary to consider the train speed level and stop plan to construct a dedicated space-time network. There are few studies in the current timetabling research and joint optimization research on the train timetable, speed level and stop plan is lacking. By solving these problems, this paper makes the following improvements to the literature:

1. A CMLST network. This network is composed of multiple space-time networks. The choice of a space-time network by the train entails the choice of a speed level and stop plan. To reduce the network scale, a network compression technology is proposed to generate the CMLST network from a MLST network.
2. A linear flow-based binary programming model. The model can describe the problem of determining the saturated timetable by maximizing the number of trains in a section. New constraints are constructed based on the proposed CMLST network, including train selection constraints and train safety constraints and so on.
3. A timing-cycle iterative optimizing method approach is combined with a greedy rule for solving the problem. We use the timing-cycle iterative optimizing method to decompose the original problem into subproblems. To improve the accuracy of the results, the subproblems are solved by the branch-and-bound algorithm using the Gurobi solver. The whole algorithm process implemented by Python.

## Problem statement and network construction

### Problem statement

**Fig 1:**
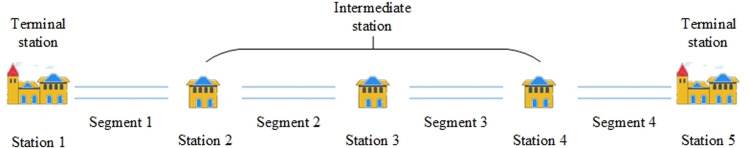
Example of a high-speed railway passenger flow section.

This paper considers a high-speed railway passenger flow section, as illustrated in Fig 1, which contains two terminal stations and several intermediate stations. The passenger flow section of a high-speed railway is the basic spatial unit of train operation. The terminal station is the station where the train starts or ends, usually with a large number of passengers boarding and alighting. For an intermediate station, the train only passes through or stops for a short period of time and does not start or end at this station. Generally, the train has to stop at a terminal station, while the train can choose to stop or not at an intermediate station.

The problem of this paper is to maximize the number of trains in the passenger flow section within a certain time horizon under the conditions of meeting the passenger demands and the constraints on the number of different types of trains and safety headway.

1. Passenger demands
  A typical passenger train scheduling process consists of the following sequential steps: passenger demand surveying, train service plan development, and train timetabling. This paper focuses on the third step. Indirectly, passenger demands are considered in this timetabling process as the number of intermediate stops.
2. Number of different types of trains
  There are many types of trains operating in the passenger flow section, which is mainly reflected by the speed level. Due to the limitation of the number of electrical multiple units (EMUs), the number of trains of a certain speed level is limited.
3. Safety headway constraints
  To ensure the safety of train operation, it is necessary to preserve a minimum time interval (headway) between trains to guarantee that there is no conflict in the train schedule.
4. Cross-line trains
  There are a large number of cross-line trains from other lines in the passenger flow section of high-speed railways. The stop plan and speed level of cross-line trains are determined by their own line, and cross-line trains generally have higher priority than the trains on the current line.

The stop plan and the speed level of the train will affect the running time of the train in the passenger flow section, thereby affecting the maximum number of trains in the passenger flow section. To maximize the number of trains, the problem studied in this paper is to determine the train stop plan, speed level and train timetable at the same time to get a global optimal solution.

### Network construction

Before describing the network, the related sets and parameters are defined in Table 2.

**Table 2:** Subscripts and parameters used in network construction.

Symbol	Definition
*S*	Set of stations
*M*	Set of intermediate stations
*E*	Set of track segments
*T*	Set of time stamps in the planning time horizon
*L*	Set of trains
*P*	Set of speed levels
*K*	Set of stop plans, *K* = {0, 1, 2,…}, where the element 0 indicates that the train will not be scheduled in the timetable
*V*	Set of vertices
*A*	Set of arcs
*A*_*o*_	Set of starting arcs
*A*_*e*_	Set of segment arcs
*A*_*m*_	Set of dwell arcs
*A*_*d*_	Set of ending arcs
*s*,*s*’	Index of stations, *s*, *s*’ ∈ *S*
*m*	Index of intermediate stations, *m* ∈ *M*
*e*	Index of track segments, *e* ∈ *E*
*t*,*t*’	Time indices, *t*, *t*’ ∈ *T*
*l*	Index of trains, *l* ∈ *L*
*p*	Index of speed levels, *p* ∈ *P*
*k*	Index of stop plans, *k* ∈ *K*
*a*	Index of arcs, *a* ∈ *A*
* $\bar{o}$ *	Dummy source for a multicommodity flow
* $\bar{d}$ *	Dummy sink for a multicommodity flow
*o*_*l*_	Origin station of train *l*
*d*_*l*_	Destination station of train *l*

#### Typical space-time network

**Fig 2:**
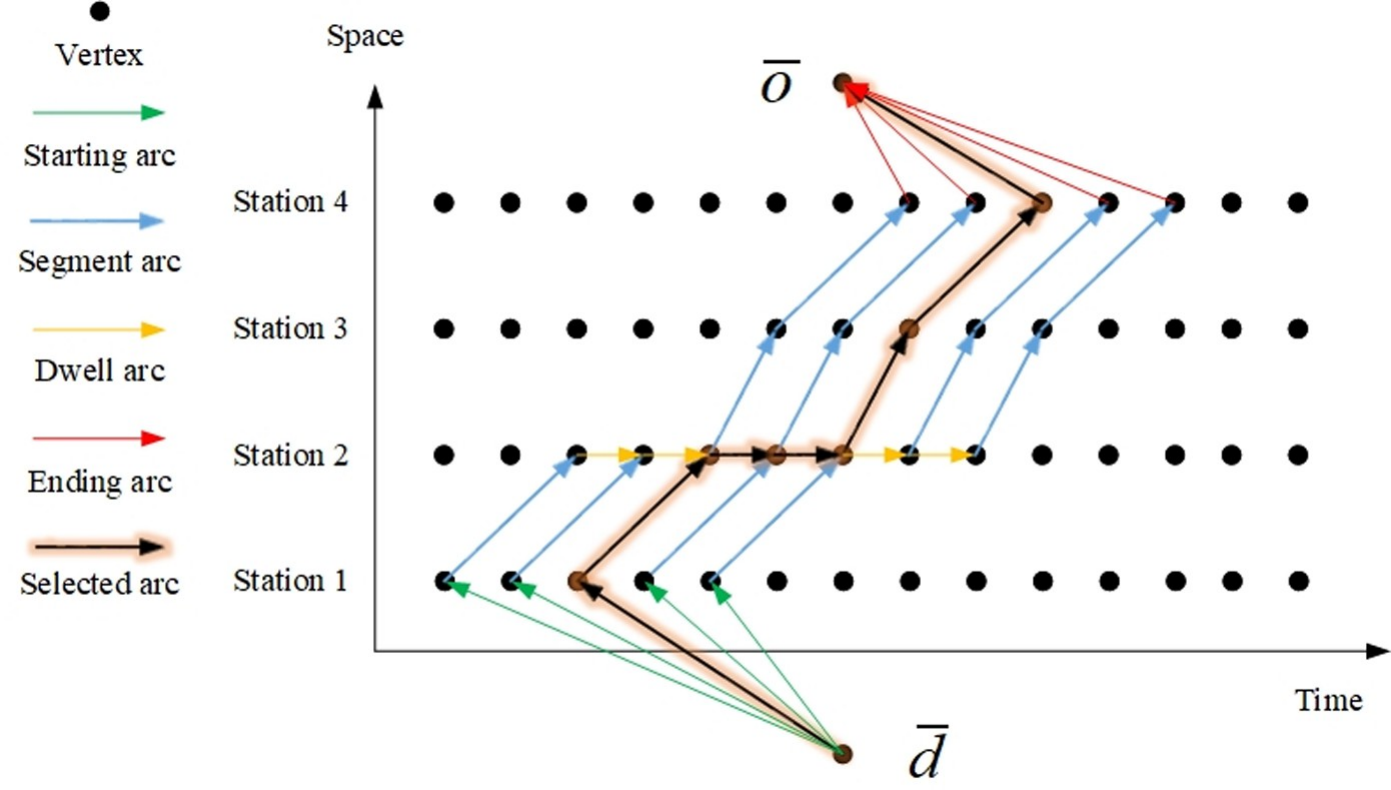
Example of a typical space-time network.

The network proposed in this paper is based on the typical space-time network. A typical space-time network is illustrated in Fig 2. The space-time network *G* = (*V*, *A*) consists of vertices and arcs. The vertex set *V* of the space-time network *G* is $V=\{\bar{o},\bar{d}\}\cup \{(s,t)|s\in S,t\in T\}$, where vertices $\bar{o}$ and $\bar{d}$ are the dummy source and dummy sink, respectively, for the multicommodity flow. A path in this network starting at $\bar{o}$ and ending at $\bar{d}$ indicates the sequence of changes in the stations of a train over time.

Arc set *A* of network *G* contains four types of arcs: *A* = *A*_*o*_ ∪ *A*_*e*_ ∪ *A*_*m*_ ∪ *A*_*d*_.

1. Starting arc set *A*_*o*_
  For the starting station *o*_*l*_ of train *l* and the start time window, there is a starting arc from the dummy source $\bar{o}$ to the point (*o*_*l*_, *t*), which means that the train starts to run from the starting station at time *t*.
2. Segment arc set *A*_*e*_
  For segment *e* and time *t*, there is a segment arc from the beginning (*s*, *t*) to the end of segment (*s*’,*t*’), which means that the train departs from station *s* at time *t* and arrives at station *s*’ at time *t*’. It should be noted that *s* and *s*’ must be two successive stations, and the difference between *t* and *t*’ is the running time of train *l* in section *e*.
3. Dwell arc set *A*_*m*_
  For intermediate station *m* and time *t*, there are dwell arcs from time *t* to time *t*+1. Each arc indicates that the train stops at the intermediate station for one time unit. The train can only select the dwell arcs of the station according to its stop plan. The number of dwell arcs selected represents the dwell time of train *l* at station *m*.
4. Ending arc set *A*_*d*_
  For the ending station *d*_*l*_ of train *l* and the end time window, there is an ending arc from the point (*d*_*l*_, *t*) to dummy sink $\bar{d}$, which indicates that the train ends its operation at time *t*.

When solving a train timetabling problem, the first step is to generate the corresponding space-time network according to the parameters of the train, that is, to determine which arcs exist in the network. Then, the process of solving the timetabling problem is the process of selecting arcs in the space-time network, and all the selected arcs form the final train timetable. In Fig 2, the starting arcs are indicated by green arrows, the segment arcs by blue arrows, the stop arcs by yellow arrows and the end arcs by red arrows. All arcs can be selected. The selected arcs are indicated by highlighted black arrows and finally form the path of the train.

#### Multilayer space-time (MLST) network construction

In contrast to the traditional train timetabling problem, the difficulty of this paper is to determine the train stop plan and speed level at the same time. However, the stop plan and speed level will affect the arcs existing in the space-time network.

For each combination of stop plans and speed levels, a different space-time network will be formed, so for each train, there will be multiple alternative space-time networks. The train must select the space-time network first and then select the arcs in the selected network. This kind of model is composed of multiple two-dimensional space-time networks, which is called an MLST network in this paper.

**Fig 3:**
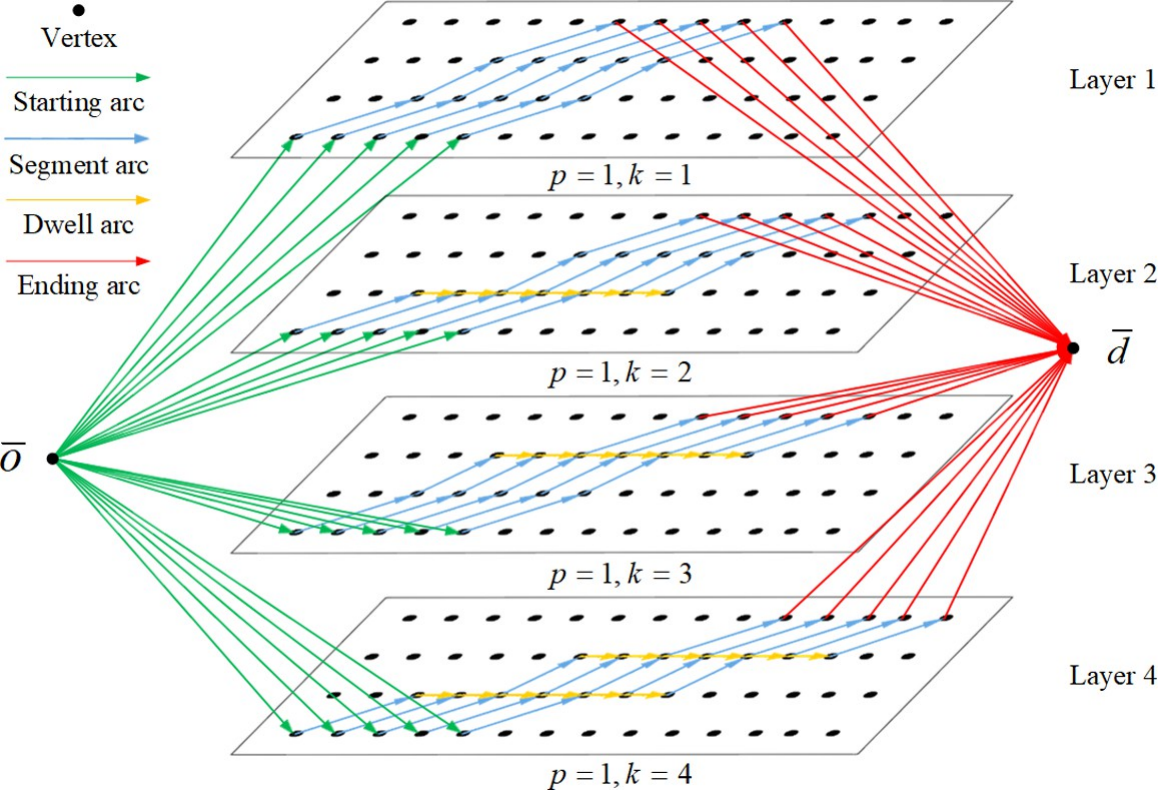
Example of an MLST (multilayer space-time) network.

An MLST network is illustrated in Fig 3. The MLST network *G* = (*V*, *A*) also consists of vertices and arcs. The vertex set *V* of the MLST network *G* is $V=\{\bar{o},\bar{d}\}\cup \{(p,k,s,t)|p\in P,k\in K,s\in S,t\in T\}$, where *p* and *k* represent the space-time network where the vertex is located. Arc set *A* of the MLST network also contains four types of arcs, but now 6 subscripts are needed to represent them, which have the form (*p*,*k*,*s*,*s*’,*t*,*t*’).

In Fig 3, there are two intermediate stations in the passenger flow section, and the train can choose to stop at an intermediate station or not. Thus, there are 4 stop plans for train *l*, *k* ∈ {1, 2, 3, 4}, so there are 4 layers of the space-time network. The layer of train selection represents the stop plan and speed level selected by the train, and the arc selected by the train in the space-time network represents the timetable of the train.

#### Compressed multilayer space-time (CMLST) network construction

The multilayer space-time network can solve the problem of determining the stop plan, speed level and train schedule at the same time. However, it is obvious that a multilayer space-time network will lead to a sharp increase in the number of arcs in the network, leading to an increase in the amount of calculation.

According to Figs 2 and 3, there are 31 arcs in the space-time network, while there are 124 arcs in the MLST network. With the increase in the number of intermediate stations and the speed level, the number of arcs in the network will increase exponentially. According to the characteristics of the problem, if an arc is selected by one train, the arcs with the same position (i.e., the same starting point and ending point) on other layers cannot be selected by other trains because this position is occupied in both time and space. This means that arcs at the same position on different layers are actually the same arc. Therefore, the arcs at the same position on different layers can be regarded as one arc, and the MLST network can be compressed into one layer, which is called a CMLST network.

**Fig 4:**
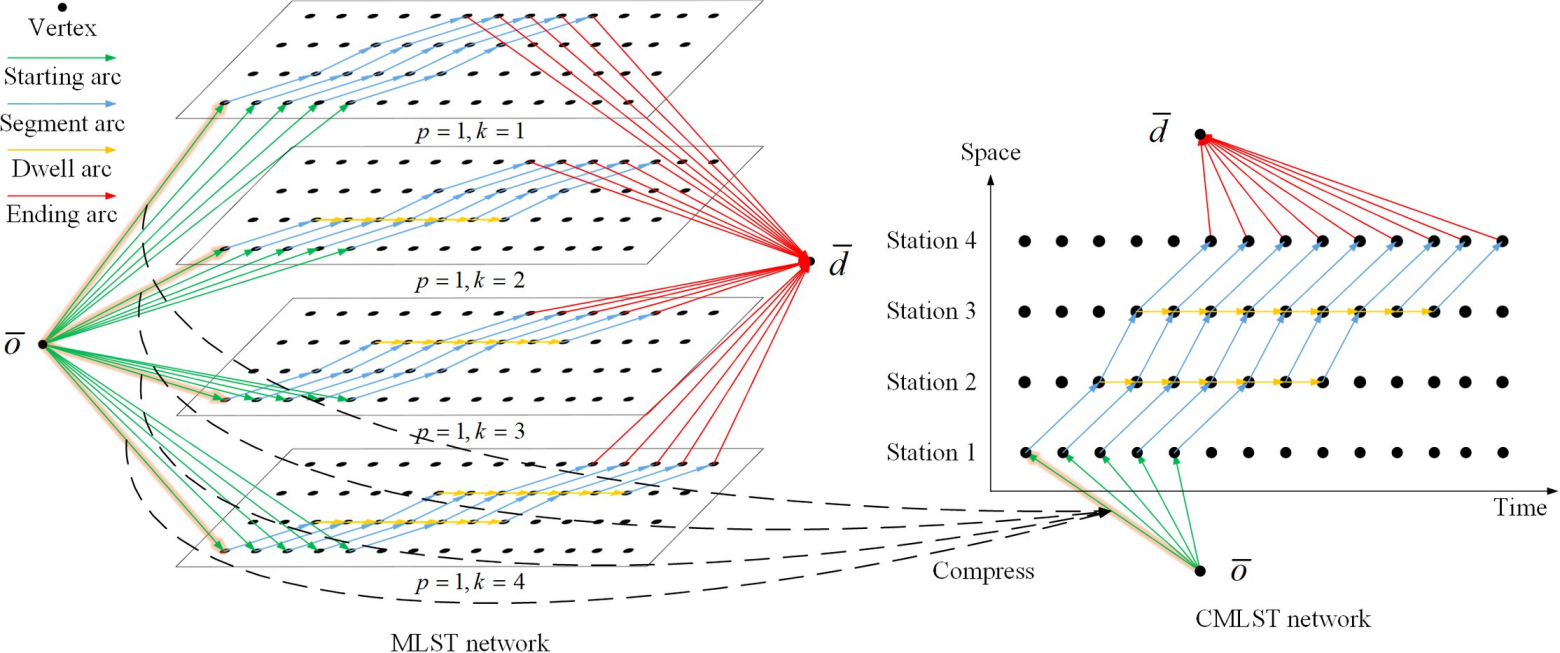
MLST network compressed into a CMLST network.

The MLST network in Fig 3 is compressed into a CMLST, as shown in Fig 4. All arcs at the same position are compressed into one. For example, the four starting arcs indicated by the highlighted green arrow in different layers are all from $\bar{o}$ to (*station* 1,0), so they can be compressed into one arc in the CMLST network. After compression, the number of arcs in the network is reduced from 124 to 49.

Although the form of the CMLST network is the same as that of the typical space-time network, it is different from the typical space-time network in that not all arcs in the network can be selected by trains. A certain type of train can only select some of the arcs. The CMLST network has a form between those of a space-time network and a space-time-state network. Compared with the space-time network, it can express more information, while compared with the space-time-state network, it can reduce the dimensionality of the network and effectively reduce the computational complexity.

## Model formulation

The optimization model aims to incorporate the train speed level and stop plan into the timetable design process considering realistic operating constraints to maximize the number of trains in the passenger flow section. Table 3 lists the notation used in the optimization model.

**Table 3:** Subscripts, parameters and decision variables used in the CMLST model.

Symbol	Definition
$\bar{K}$	Subset of *K* without element 0
$\bar{L}$	Set of cross-line trains
$\bar{A}$	Set of arcs occupied by cross-line trains, a subset of *A*
*t*”,*t*”‘	Time indices, *t*, *t’* ∈ *T*
*α*_*s*_	Additional starting time of trains at station s
*β*_*s*_	Additional stopping time of trains at station s
* $\tau_{f}^{s}$ *	Minimum headway time between the departure times of two trains from the same station *s*
$\tau_{t}^{s}$	Minimum headway time between the times of two trains passing through the same station *s*
* $\tau_{d}^{s}$ *	Minimum headway time between the times of two trains arriving at the same station *s*
* $\tau_{ft}^{s}$ *	Minimum headway time between the time of the previous train departing from station *s* and the next train passing through station *s*
* $\tau_{tf}^{s}$ *	Minimum headway time between the time of the previous train passing through station *s* and the next train departing from station *s*
* $\tau_{dt}^{s}$ *	Minimum headway time between the time of the previous train arriving at station *s* and the next train passing through station *s*
* $\tau_{td}^{s}$ *	Minimum headway time between the time of the previous train passing through station *s* and the next train arriving at station *s*
*u*_*m*_	Service frequency demand of intermediate station *m*
*g*_*m*_	Minimum stop time of intermediate station *m*
*h*_*m*_	Maximum stop time of intermediate station *m*
*c*_*p*_	Maximum number of trains with speed level *p*
*w*_*p*_	Maximum number of consecutive trains with speed level *p*
* $\rho_{s}^{k}$ *	A 0–1 parameter that is equal to 1 when trains with stop plan *k* stop at station *s* and 0 otherwise
* $r_{e}^{p}$ *	Travel time of a train with speed level *p* in section *e*
${\tilde{\text{r}}}_{e}^{p,k}$	Travel time of a train with speed level *p* in section *e*, including the additional starting time and additional stopping time
$x_{l}^{p,k,a}$	A binary variable that is equal to 1 when arc *a* is selected by train *l*, which chooses speed level *p* and stop plan *k*, and is equal to 0 otherwise
$y_{l}^{p,k}$	A binary variable that is equal to 1 when train *l* chooses speed level *p* and stop plan *k* and 0 otherwise

### Model notations

#### Objective function and constraints

*Objective function*. Within the CMLST network framework, the objective function for maximizing the total number of trains in the passenger flow section is stated in Eq (1).

$$z=max\underset{l\in L,p\in P,k\in \bar{K}}{\sum }y_{l}^{p,k}$$ (1)

Objective function (1) aims to maximize the number of trains for which stop plan *k* is not 0 (i.e., the trains are scheduled in the timetable).

*Flow balance constraint*. Since the model proposed in this paper is different from the typical space-time network model, the flow balance constraints of the model should be modified accordingly. To depict a feasible train path in the CMLST network, a set of flow balance constraints is constructed as follows:

$$\underset{a\in \{\left(\bar{o},s,t,t\right)\in A_{o}|s=o_{l}\}}{\sum }x_{l}^{p,k,a}=y_{l}^{p,k}\;\forall l\in L,p\in P,k\in \bar{K}$$ (2)

$$\underset{a\in \{\left(s,\bar{d},t,t\right)\in A_{d}|s=d_{l}\}}{\sum }x_{l}^{p,k,a}=y_{l}^{p,k}\;\forall l\in L,p\in P,k\in \bar{K}$$ (3)

$$\underset{a\in \left\{\left(s,s',t,t'\right)\in A\right\}}{\sum }x_{l}^{p,k,a}=\underset{a'\in \left\{\left(s',s,t',t\right)\in A\right\}}{\sum }x_{l}^{p,k,a'}\;\forall l\in L,p\in P,k\in \bar{K},\left(s,t\right)\in V$$ (4)

Constraint (2) means that for each individual train *l*, if train *l* selects speed level *p* from set *P* and stop plan *k* from set $\bar{K}$, then $y_{l}^{p,k}=1$ and there should be one and only one arc selected from all the starting arcs of train *l* from $\bar{o}$ to (*o*_*l*_,*t*) Otherwise, if $y_{l}^{p,k}=0$ for all *p* ∈ *P* and $k\in \bar{K}$, then there should be no arc selected from all the starting arcs of train *l*.

Constraint (3) means that for each individual train *l*, if train *l* selects speed level *p* from set *P* and stop plan *k* from set $\bar{K}$, then $y_{l}^{p,k}=1$ and there should be one and only one arc selected from all the ending arcs of train *l* from (*d*_*l*_, *t*) to $\bar{d}$. Otherwise, if $y_{l}^{p,k}=0$ for all *p* ∈ *P* and $k\in \bar{K}$, then no arc should be selected from the ending arcs of train *l*.

Constraint (4) means that for all vertices (*s*, *t*) ∈ *V*, the number of arcs from (*s*, *t*) to (*s*’, *t*’) should equal the number of arcs from (*s*’, *t*’) to (*s*, *t*).

These constraints ensure that when a train is scheduled, it will start from the dummy origin and end at the dummy sink after a series of network vertices, and the selected arcs will finally form a complete path.

*Train selection constraint*. The selection of trains in set *L* is not arbitrary. It must not only meet the service frequency demand of the stations but also consider the limitation of the number of consecutive trains and the resources of the EMU. A set of train selection constraints is constructed as follows:

$$\underset{p\in P,k\in K}{\sum }y_{l}^{p,k}=1\;\forall l\in L$$ (5)

$$\underset{l\in L,p\in P,k\in \bar{K}}{\sum }y_{l}^{p,k}\cdot \rho_{m}^{k}\geq u_{m}\;\forall m\in M$$ (6)

$$\underset{l\in L,k\in \bar{K}}{\sum }y_{l}^{p,k}\leq c_{p}\;\forall p\in P$$ (7)

$$\underset{l\in L,k\in \bar{K},a\in \{\left(e,t,t^{\prime }\right)|t^{\prime }\in [t,t+\tau_{f}^{s}\cdot \left(w_{p}+1\right)\}}{\sum }x_{l}^{p,k,a}\leq w_{p}\;\forall p\in P,e\in E,t\in T$$ (8)

Constraint (5) means that for all trains in *L*, a combination of a speed level *p* and stop plan *k* must be selected, and if *k* = 0 in the selected combination, it means that the train will not be scheduled in the timetable.

Constraint (6) means that for intermediate station *m*, the number of trains stopping at station *m* ($\rho_{m}^{k}=1$) should be greater than or equal to the demand of station *m*.

Constraint (7) means that the number of trains with speed level *p* should be less than the maximum number of EMU resources.

Consecutive trains with the same speed level can improve the utilization rate of the capacity, but it will lead to a strong homogeneity of trains, which will cause concentrated train stops and long waiting times of overtaken low-speed trains at the stations. Constraint (8) limits the number of consecutive trains with the same speed level *p* in section *e*.

*Train dwell constraint*. The dwell of train *l* at station *m* should be consistent with the selected stop plan *k*, and the stop time should be between the maximum stop time and the minimum stop time of station *m*. At the same time, the additional starting and stopping time of train *l* at station *m* should be considered. A set of train dwell constraints is constructed as follows:

$$\underset{a\in \left\{\left(m,m,t,t^{\prime }\right)\in A_{m}\right\}}{\sum }x_{l}^{p,k,a}=0\;\forall l\in L,p\in P,k\in \bar{K},m\in M|\rho_{m}^{k}=0$$ (9)

$$\underset{a\in \left\{\left(m,m,t,t^{\prime }\right)\in A_{m}\right\}}{\sum }x_{l}^{p,k,a}\geq y_{l}^{p,k}\cdot g_{m}\;\forall l\in L,p\in P,k\in \bar{K},m\in M|\rho_{m}^{k}=1$$ (10)

$$\underset{a\in \left\{\left(m,m,t,t^{\prime }\right)\in A_{m}\right\}}{\sum }x_{l}^{p,k,a}\leq y_{l}^{p,k}\cdot h_{m}\;\forall l\in L,p\in P,k\in \bar{K},m\in M|\rho_{m}^{k}=1$$ (11)

$$\underset{a\in \{\left(s,s^{\prime },t,t^{\prime }\right)\in A_{e}|s\geq o_{l},s^{\prime }\leq d_{l},t^{\prime }=t+{\tilde{\text{r}}}_{e}^{p;k}\}}{\sum }x_{l}^{p,k,a}=y_{l}^{p,k}\;\forall l\in L,p\in P,k\in \bar{K},e\in E$$ (12)

Constraint (9) means that if train *l* with stop plan *k* does not stop at intermediate station *m* ($\rho_{m}^{k}=0$), then train *l* cannot select the dwell arcs of intermediate station *m*.

Constraints (10) and (11) mean that if train *l* with stop plan *k* stops at intermediate station *m* ($\rho_{m}^{k}=1$), then the number of dwell arcs of intermediate station *m* selected by train *l* should be greater than or equal to the shortest stop time *g*_*m*_ and less than or equal to the longest stop time *h*_*m*_.

**Fig 5:**
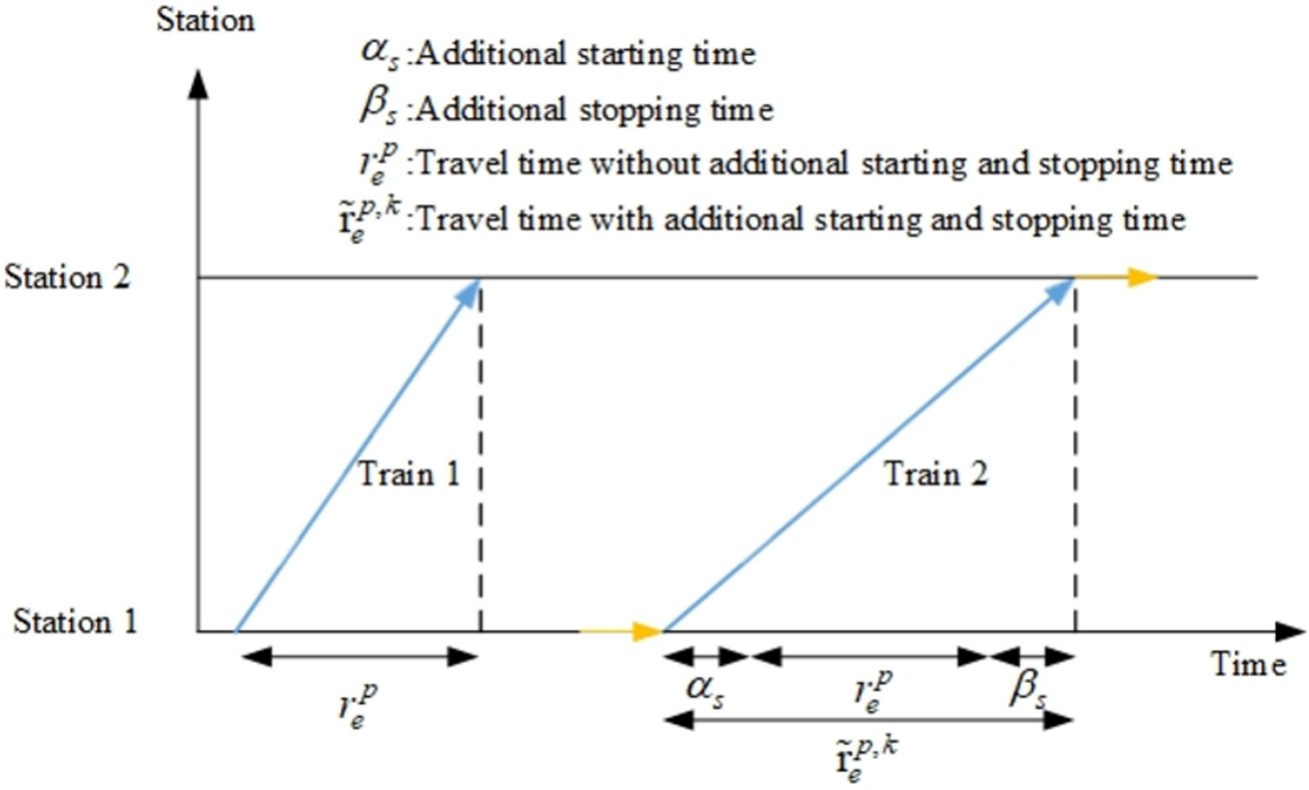
Example of additional starting and stopping time.

The dwell of train *l* at station *m* needs to include the process of starting and braking, which will affect the travel time in segment *e*. As shown in Fig 5, Train 1 does not stop at either Station 1 or Station 2, while Train 2 stops at both Station 1 and Station 2. Therefore, the travel time of Train 2 in section *e* is equal to the travel time of Train 1 $r_{e}^{p}$ plus the additional starting time *α*_*s*_ at Station 1 and the additional stopping time *β*_*s*_ at Station 2.

The purpose of constraint (12) is to ensure that the difference between *t* and *t*’ of the selected arc (*s*,*s*’,*t*,*t*’) is equal to the standard travel time plus the additional starting and stopping time.

*Train safety (time headway) constraint*. To guarantee the safety of high-speed railway operation, there is a minimum time headway between the departure, arrival and passing through of two consecutive trains at the same station. In this paper, seven kinds of train safety constraints are considered in the CMLST network model, as follows:

$$\underset{l\in L,p\in P,k\in \bar{K},a\in \{\left(s,s^{\prime },t^{\prime },t^{\prime \prime }\right)\in A_{e}|t^{\prime }\in \left[t,t+\tau_{f}^{s}\right],\rho_{s}^{k}=1\}}{\sum }x_{l}^{p,k,a}\leq 1\;\forall \left(s,s^{\prime }\right)\in E,t\in T$$ (13)

$$\underset{l\in L,p\in P,k\in \bar{K},a\in \{\left(s,s^{\prime },t^{\prime },t^{\prime \prime }\right)\in A_{e}|t^{\prime \prime }\in \left[t,t+\tau_{d}^{s}\right],\rho_{s'}^{k}=1\}}{\sum }x_{l}^{p,k,a}\leq 1\;\forall \left(s,s^{\prime }\right)\in E,t\in T$$ (14)

$$\underset{l\in L,p\in P,k\in \bar{K},a\in \{\left(s,s^{\prime },t^{\prime },t^{\prime \prime }\right)\in A_{e}|t^{\prime }\in \left[t,t+\tau_{t}^{s}\right],\rho_{s}^{k}=0\}}{\sum }x_{l}^{p,k,a}\leq 1\;\forall \left(s,s^{\prime }\right)\in E,t\in T$$ (15)

$$\begin{array}{r} \underset{l\in L,p\in P,k\in \bar{K},a\in \{\left(s,s^{\prime },t,t^{\prime }\right)\in A_{e}|\rho_{s}^{k}=1\}}{\sum }x_{l}^{p,k,a}\text{+}\underset{l\in L,p\in P,k\in \bar{K},a'\in \{\left(s,s^{\prime },t^{\prime \prime },t^{\prime \prime \prime }\right)\in A_{e}|\rho_{s}^{k}=0,t^{\prime \prime }\in \left[t,t+\tau_{ft}^{s}\right]\}}{\sum }x_{l}^{p,k,a'}\leq 1\;\\ \forall \left(s,s^{\prime }\right)\in E,t\in T \end{array}$$ (16)

$$\begin{array}{r} \underset{l\in L,p\in P,k\in \bar{K},a\in \{\left(s,s^{\prime },t,t^{\prime }\right)\in A_{e}|\rho_{s}^{k}=0\}}{\sum }x_{l}^{p,k,a}+\underset{l\in L,p\in P,k\in \bar{K},a'\in \{\left(s,s^{\prime },t^{\prime \prime },t^{\prime \prime \prime }\right)\in A_{e}|\rho_{s}^{k}=1,t^{\prime \prime }\in \left[t,t+\tau_{tf}^{s}\right]\}}{\sum }x_{l}^{p,k,a'}\leq 1\\ \forall \left(s,s^{\prime }\right)\in E,t\in T \end{array}$$ (17)

$$\begin{array}{r} \underset{l\in L,p\in P,k\in \bar{K},a\in \{\left(s,s^{\prime },t,t^{\prime }\right)\in A_{e}|\rho_{s'}^{k}=1\}}{\sum }x_{l}^{p,k,a}+\underset{l\in L,p\in P,k\in \bar{K},a'\in \{\left(s,s^{\prime },t'',t'''\right)\in A_{e}|\rho_{s'}^{k}=0,t'''\in \left[t',t'+\tau_{dt}^{s}\right]\}}{\sum }x_{l}^{p,k,a'}\leq 1\\ \forall \left(s,s^{\prime }\right)\in E,t'\in T \end{array}$$ (18)

$$\begin{array}{r} \underset{l\in L,p\in P,k\in \bar{K},a\in \{\left(s,s^{\prime },t,t^{\prime }\right)\in A_{e}|\rho_{s'}^{k}=0\}}{\sum }x_{l}^{p,k,a}+\underset{l\in L,p\in P,k\in \bar{K},a'\in \{\left(s,s^{\prime },t'',t'''\right)\in A_{e}|\rho_{s'}^{k}=1,t'''\in \left[t,t+\tau_{td}^{s}\right]\}}{\sum }x_{l}^{p,k,a'}\leq 1\\ \forall \left(s,s^{\prime }\right)\in E,t'\in T \end{array}$$ (19)

Constraint (13) means that for segment *e* = (*s*,*s*’), time *t* and trains that depart from station *s* in the time range $t'\in [t,t+\tau_{f}^{s}]$ and arrive at station *s*’ at time *t*”, at most one segment arc *a* = (*s*,*s*’,*t*,*t*”) can be selected in order to ensure the minimum headway time between two trains departing from the same station *s* in succession.

Constraint (14) means that for segment *e* = (*s*, *s*’), time *t* and trains that arrive at station *s*’ in the time range $t''\in [t,t+\tau_{d}^{s}]$ and depart from station *s* at time *t*’, at most one segment arc *a* = (*s*,*s*’,*t*’,*t*”) can be selected in order to ensure the minimum headway time between two trains arriving at the same station *s*’ in succession.

Constraint (15) means that for segment *e* = (*s*, *s*’), time *t* and trains that pass through station *s* in the time range $t'\in [t,t+\tau_{t}^{s}]$ and arrive at station *s*’ at time *t*”, at most one segment arc *a* = (*s*,*s*’,*t*’,*t*”) can be selected in order to ensure the minimum headway time between two trains passing through the same station *s* in succession.

Constraint (16) means that for segment *e* = (*s*, *s*’), time *t* and two trains, if the first train departs from station *s* at time *t*, then the second train, which passes through station *s* at time *t*”, cannot select arcs in the time range $\left[t,t+\tau_{ft}^{s}\right]$. Otherwise, at most one arc can be selected by the latter train to ensure the minimum headway time between the two trains.

Constraint (17) means that for segment *e* = (*s*, *s*’), time *t* and two trains, if the first train passes through station *s* at time *t*, then the second train, which departs from station *s* at time *t*”, cannot select arcs in the time range $\left[t,t+\tau_{tf}^{s}\right]$. Otherwise, at most one arc can be selected by the latter train.

Constraint (18) means that for segment *e* = (*s*, *s*’), time *t*’ and two trains, if the first train arrives at station *s*’ at time *t*’, then the second train, which passes through station *s*’ at time *t*”‘, cannot select arcs in the time range $\left[t',t'+\tau_{dt}^{s}\right]$. Otherwise, at most one arc can be selected by the latter train.

Constraint (19) means that for segment *e* = (*s*, *s*’), time *t*’ and two trains, if the former train passes through station *s*’ at time *t*’, then the latter train, which arrives at station *s*’ at time *t*”‘, cannot select arcs in time range $\left[t',t'+\tau_{td}^{s}\right]$. Otherwise, at most one arc can be selected by the latter train.

*Train overtaking constraint*. Train overtaking can only occur in a station, not in a segment; otherwise, there will be conflict.


$$\begin{array}{r} \underset{l\in L,p\in P,k\in \bar{K}|t^{\prime }=t+{\tilde{\text{r}}}_{e}^{p,k}}{\sum }x_{l}^{p,k,a}+\underset{l^{\prime }\in L,p^{\prime }\in P,k^{\prime }\in \bar{K},a^{\prime }\in \{\left(s,s^{\prime },t^{\prime \prime },t^{\prime \prime \prime }\right)\in A_{e}|t^{\prime \prime }>t,t^{\prime \prime \prime }<t^{\prime },t^{\prime \prime \prime }=t^{\prime \prime }+{\tilde{\text{r}}}_{e}^{p,k}\}}{\sum }x_{l^{\prime }}^{p^{\prime },k^{\prime },a^{\prime }}\leq 1\;\\ \forall a\in \left\{\left(s,s^{\prime },t,t^{\prime }\right)\in A_{e}\right\} \end{array}$$ (20)

Constraint (20) means that if segment arc *a* = (*s*,*s*’,*t*,*t*’) is selected by train *l*, then the arcs *a*’ = (*s*,*s*’,*t*’,*t*”) crossing arc *a* in the segment cannot be selected by other trains.

*Cross-line train constraint*. For high-speed railway passenger flow sections with long mileage, there are usually some trains from other lines called cross-line trains, which usually have a higher priority than the trains in the research section. In this model, cross-line train *l* with speed level $\bar{p}$ and stop plan $\bar{k}$ can be scheduled in advance by setting the relevant variable to 1, as shown in Eq (21). The arcs occupied by the cross-line trains in the network cannot be selected by other trains again.

$$x_{l}^{\bar{p},\bar{k},a}=1\;\forall l\in \bar{L},a\in \bar{A}$$ (21)

*Binary variable constraint*. There are also binary definitional constraints for variables $x_{l}^{p,k,a}$ and $y_{l}^{p,k}$.


$$x_{l}^{p,k,a}\in \{0,1\}\;\forall l\in L,p\in P,k\in K,a\in A$$ (22)

$$y_{l}^{p,k}\in \text{\{0},1\}\forall l\in L,p\in P,k\in K$$ (23)

## Timing cycle iterative optimizing method

The CMLST network can reduce the arcs of the network and the calculation time significantly. However, due to the complexity of railway systems, the scale of real-world problems is often beyond control. For the space-time network model, the variable size is equal to the number of arcs in the network. When the time dimension and space dimension of the case increase linearly, the number of available arcs in the network will increase exponentially, resulting in very complex problem solving. Therefore, we need to find an effective method to address this situation.

**Fig 6:**
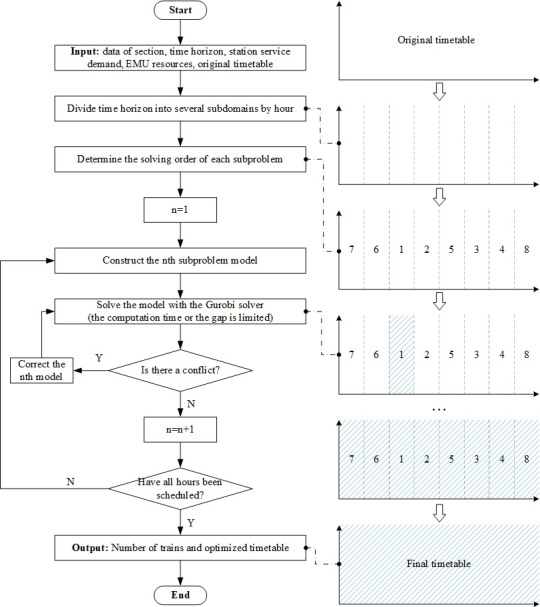
Flow diagram for the timing-cycle iterative optimizing method.

The timing-cycle iterative optimizing method is often used in a large-scale railway timetabling problem. The idea of the method is as follows: divide the time horizon into several subdomains, and schedule the trains in each subdomain in turn. When a subdomain is saturated, it is no longer possible to add new trains in this subdomain. Therefore, when a subdomain is scheduled, only the arcs of the current subdomain need to be constructed, reducing the scale of the problem. In the calculation of a subdomain, it is necessary to consider the influence of trains in other subdomains and to take the trains that may conflict as the known input to ensure that there is no conflict. In this way, the original problem is divided into several subproblems, and each subproblem can find the optimal solution in a limited time. After all the subproblems are solved, the solutions of all the subproblems are combined into a complete solution set, and the solution of the original problem is obtained. The flow diagram for the timing-cycle iterative optimizing method is presented in Fig 6.

For the determination of the order of the subproblems, this paper introduces the idea of greedy rules. It is known that trains scheduled in one subdomain will affect the adjacent subdomains. Since our goal is to maximize the number of trains in the timetable, we should solve the most intense time domain first. In terms of specific quantitative indicators, if there are more stop demands in a time domain, the order of this time domain should be higher.

## Case study

We take the real-world case of the Beijing South to Jinan West passenger flow section of the Beijing-Shanghai high-speed railway as an example, which is one of the most important high-speed rail lines in China.

### Data input

In terms of infrastructure, there are 6 stations, 5 segments and 4 intermediate stations in the Beijing South-Jinan West passenger flow section, and the total mileage is 419 kilometers. This case considers a full day of operation, from 6:00 to 24:00. The number of available trains is set to 216 (12 trains per hour). The details are shown in Table 4.

**Table 4:** Infrastructure-related settings.

Parameter	Value
Planning horizon	6:00–24:00
Number of stations (size of *S*)	6
Number of intermediate stations (size of *M*)	4
Number of segments (size of *E*)	5
Number of time stamps (size of *T*)	1080
Number of trains available (size of *L*)	216 (12 per hour)
Number of stop plans (size of *K*)	17 (2^size of *M*^ +1)

According to the data obtained from the survey of railway company. There are two speed levels of trains in the studied section, namely, 300 km/h and 350 km/h. The travel time of trains at the two speed levels in each segment and the additional starting and stopping time of each station are shown in Table 5.

**Table 5:** Travel time and additional time of trains in each segment.

Station	Travel time $r_{e}^{p}$ (min)		Additional time (min)	
	350 km/h	300 km/h	*α*_*s*_	*β*_*s*_
Beijing South			2	2
	15	16		
Langfang			3	3
	11	13		
Tianjing South			3	3
	16	18		
Cangzhou West			3	3
	18	20		
Dezhou East			3	3
	17	19		
Jinan West			3	3

The minimum headway time and stop time at each station are the same, as shown in Table 6.

**Table 6:** Headway time and stop time of stations.

Parameter	Value
Minimum headway time between the times of two trains departing from the same station *s* ($\tau_{f}^{s}$)	5 minutes
Minimum headway time between the times of two trains passing through the same station *s* ($\tau_{t}^{s}$)	4 minutes
Minimum headway time between the times of two trains arriving at the same station *s* ($\tau_{d}^{s}$)	5 minutes
Minimum headway time between the time of the previous train departing from station *s* and the next train passing through station *s* ($\tau_{ft}^{s}$)	5 minutes
Minimum headway time between the time of the previous train passing through station *s* and the next train departing from station *s* ($\tau_{tf}^{s}$)	2 minutes
Minimum headway time between the time of the previous train arriving at station *s* and the next train passing through station *s* ($\tau_{dt}^{s}$)	3 minutes
Minimum headway time between the time of the previous train passing through station *s* and the next train arriving at station *s* ($\tau_{td}^{s}$)	5 minutes
Minimum stop time of intermediate station *m* (*g*_*m*_)	2 minutes
Maximum stop time of intermediate station *m* (*h*_*m*_)	10 minutes

We used Gurobi 9.1 and the timing-cycle iterative optimizing method (implemented in Python 3.7) to solve the CMLST model. In the process of problem solving, Python’s function is to implement iterative optimization method proposed in section 5, including processing input data, dividing the problem into subproblems and integrating the results of subproblems. The function of Gurobi solver is to solve subproblems in turn. The specific method used is the branch pricing method. For the Gurobi solver, we can set the parameters according to the characteristics of the problem to improve the calculation speed, as shown in Table 7. The MIPGap refers to the max relative difference between the solution and the theoretical optimal solution. The value of MIPGap affects the calculation time and the quality of solution. For the large-scale optimization problem in this paper, the theoretical optimal solution usually cannot be found in an acceptable time. Therefore, it is necessary to find an appropriate MIPGap value to obtain a satisfactory solution in an acceptable time. Through a large number of numerical experiments, we find that it is appropriate to set the value to 0.1, that is, to ensure that the relative difference between the obtained solution and the optimal solution is within 10%.

**Table 7:** Parameter settings of Gurobi.

Parameter	Value
MIPGap	0.1
MIPGapAbs	1
Method	2
MIPFocus	1
Presolve	2
VarBranch	3
ImproveStartTime	100

### Computational results

**Fig 7:**
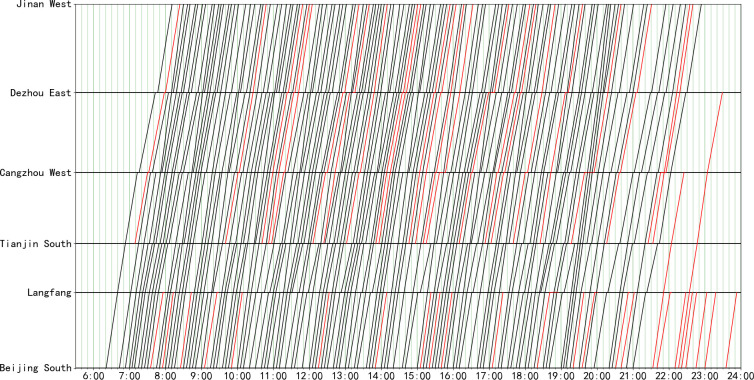
Real-world timetable of the Beijing-Shanghai high-speed rail line (152 trains).

**Fig 8:**
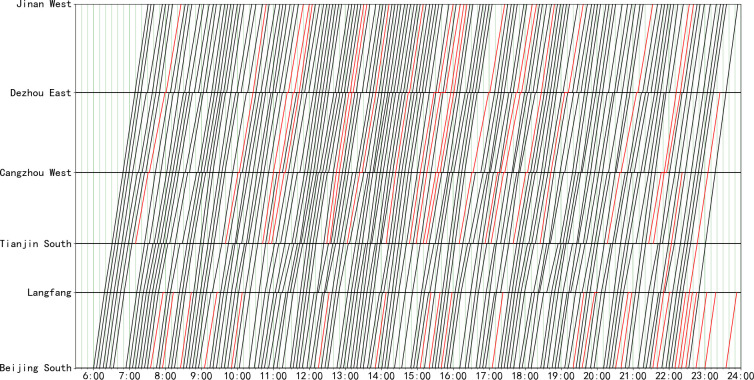
Optimized timetable for the Beijing-Shanghai high-speed rail line (171 trains).

Given the real-world schedule of the Beijing-Shanghai high-speed rail line shown in Fig 7, we generate an optimized real-world train timetable in Fig 8. The overall total train number is increased from 152 trains to 171 trains (with an equivalent capacity enhancement of approximately 12.5%). Cross-line trains are indicated by red lines, and some adjustments can be made for times when the capacity is too tight. There is no conflict between trains, and all the kinds of constraints set by the model are satisfied, which proves that the model is effective.

The calculation results for each hour are shown in Table 8. As the busiest high-speed rail line in China, the capacity utilization rate of the case section is 100% in 11 hours of the day. The available trains are all in periods of less passenger flow, which indicates that the utilization of capacity in the current transportation plan is close to saturation, so it is necessary to take further measures to strengthen the capacity or optimize the transportation organization. In terms of the calculation process, the gap value of the solution in each period can be controlled to below 10%, which indicates that the quality of the solution is good.

**Table 8:** Comparison of the number of trains per hour before and after optimization.

Time horizon	Before	After	Difference	Gap
6:00–7:00	4	9	5	1.43%
7:00–8:00	11	11	0	2.60%
8:00–9:00	10	10	0	8.38%
9:00–10:00	10	10	0	9.87%
10:00–11:00	11	11	0	9.54%
11:00–12:00	9	9	0	3.39%
12:00–13:00	11	11	0	8.67%
13:00–14:00	10	10	0	7.01%
14:00–15:00	10	11	1	5.82%
15:00–16:00	12	12	0	9.88%
16:00–17:00	9	9	0	7.59%
17:00–18:00	10	11	1	9.25%
18:00–19:00	8	9	1	8.11%
19:00–20:00	10	10	0	7.35%
20:00–21:00	7	10	3	4.83%
21:00–22:00	3	8	5	7.84%
22:00–23:00	5	8	3	0.00%
23:00–24:00	2	2	0	0.00%
Total	152	171	19	/

## Conclusion

This paper studies a railway capacity increasing problem that integrates train timetabling with the train speed level and stop plan. The capacity increasing problem is solved as a joint optimization model timetable saturation problem. The saturated timetable for capacity increasing purposes has the maximum number of trains given the infrastructure- and passenger demand-related aspects. We use a compressed multilayer space-time network to model this optimization problem and propose integer programming that maximizes the number of trains in the railway sections. A timing-cycle iterative optimizing method is proposed to solve this problem efficiently. According to the case test, the integration problem is managed well by the iterative approach proposed.

Academically, this paper extends the current space-time network-based modeling method to a multilayer space-time network, and compression technology is proposed to reduce the number of arcs in the network. This is a beneficial proposal. In practical applications, the train timetable generated by the CMLST model can intuitively show the carrying capacity and its usage in each hour, indicating at which periods more trains can be added and providing a reference schedule for the railway company.

In future research, we will consider designing a multiobjective model based on the approach proposed in this paper to find a Pareto solution that can balance the total train number, total travel time, total dwell time and so on. For larger-scale real-world problems, it may be necessary to design heuristic methods to reduce the number of combinations of speed levels and stop plans according to the specific passenger demand.

## Supporting information

S1 Data (XLSX)
